# Recovery from Bite of a Rattlesnake

**Published:** 1874-01

**Authors:** W. F. C. Beattie

**Affiliations:** Cornwall, N. Y.


*Recovery from Bite of a Rattlesnake.* By W. F. C. Beattie, M.D., Cornwall, N. Y.

At 7 A. M., June 28th, Joseph Hulse, aged fifty-two years, (living a hermit's life in the mountains), seized a live rattlesnake by the neck with the naked hand. The snake, having play enough of head, inserted his fangs at the second joint of the right index-finger. At 10 o'clock, A. M., after walking three miles in the hot sun, Hulse presented himself, pale, trembling, and cadaverous, reeling, when attempting to walk, like a drunken man, and frequently falling as though dead, but immediately recovering muscular power when in a prone position, and then able again to rise. I assisted him to a neighboring barn, and placed him on a bed of straw. His pulse was 160, with frequent intermissions. His hand and arm were fearfully swollen, and there was ecchymosis of the arm and the anterior part of the chest. The mind was somewhat incoherent. Fearful as seemed the train of symptoms, I immediately laid open the wounded finger through an inch of gangrenous flesh at the seat of injury. He then swallowed half a pint of Bourbon whisky, and repeated the dose every five minutes until he had taken one quart. The first effect was to reduce the pulse to 100 per minute, and tranquillize the mind. Then profound inebriation ensued. The arm was laid in a large poultice of mud, the first effect being to cause loud complaints of pain at the seat of injury, although there had been no sensation on using the lance. After four hours of stupor the patient awoke, when an empiric, in my absence, cleansed his arm and very tightly bandaged fingers and arm above the elbow. In this condition he was conveyed to the poor-master. Word was left that I need not see him until morning, when I found, to my horror, that the circulation was so completely cut off, that on removing the bandage, incipient gangrene of the sore finger had occurred. Vitality, however, was restored, and the finger got well after a sloughing process. I now ordered milk, with the addition of a teaspoonful of whisky, the whisky to be increased to a tablespoonful when the stomach would bear it, and to be taken *ad libitum.* I then ordered sesquicarbonate of ammonia, grs. x, every two hours, and administered three compound cathartic pills. In the afternoon an officious friend applied a white-ash bark ligature at the middle of the arm so tightly that serum exuded, which a bystander warned me not to interfere with, as it was the "poison coming out." There was now obstinate hemorrhage from the gums and from the wound in the finger, which was checked by perchloride of iron, and an elevated position of the arm. The blood had lost its property of coagulating, and lay for hours, in the hot sun, fluid as turpentine. This seemed proof positive that the poison had permeated the system. After a few days of the above treatment, I ordered tonics, and four weeks later the man was at work. I think we may learn from this case that a rattlesnake-bite, although the poison has impregnated the system, is not necessarily fatal.—*New York Medical Jour.*

